# Can glass polyalkenoate (glass-ionomer) dental cements be considered bioactive? A review

**DOI:** 10.1016/j.heliyon.2024.e25239

**Published:** 2024-02-02

**Authors:** John W. Nicholson, Sharanbir K. Sidhu, Beata Czarnecka

**Affiliations:** a Bluefield Centre for Biomaterials, 152-160 City Road, London EC1V 2NX, UK *and* Dental Physical Sciences, Institute of Dentistry, Barts & The London School of Medicine and Dentistry, Queen Mary University of London, Mile End Road, London, E1 4NS, UK; b Centre for Oral Bioengineering, Institute of Dentistry, Barts & The London School of Medicine and Dentistry, Queen Mary University of London, Turner Street, London E1 2AD, UK; c Department of Biomaterials and Experimental Dentistry, Poznań University of Medical Sciences, Ul. Bukowska 70, 60-812 Poznań, Poland

**Keywords:** Glass-ionomer, Polyalkenoate, Bioactivity, Bioactive glass, Ion-release, pH change, Literature review

## Abstract

**Objectives:**

This paper reviews the chemical behaviour of glass polyalkenoate (glass-ionomer) dental cements, both conventional and resin-modified, in contact with natural tissues, with the aim of determining whether these materials can be considered to be bioactive.

**Data:**

Relevant papers describing the behaviour of bioactive glasses and ceramics, and glass-ionomer (glass polyalkenoate) cements have been identified using PubMed and Science Direct. This has allowed a comparison to be made between the behaviour of glass-ionomers and the speciality glasses and ceramics that are widely classified as bioactive, a designation considered valid for over fifty years. More recent papers concerning bioactive metals and polymers have also been studied and both *in vitro* and *in vivo* studies are included.

**Sources:**

Have included general papers on the chemistry and biological behaviour of bioactive glasses and ceramics, as well as papers on glass-ionomers dealing with (i) ion release, (ii) bonding to the surface of teeth, (iii) influence on surrounding pH and (iv) interaction with bone.

**Conclusion:**

The literature shows that glass-ionomers (glass polyalkenoates) have three types of behaviour that are similar to those of bioactive glasses as follows: Formation of direct bonds to living tissue (teeth and bones) without fibrous capsule; release of biologically beneficial ions; and change of the local pH. However, in *in vitro* tests, they do not cause calcium phosphate to precipitate from solutions of simulated body fluid, SBF. Despite this, studies show that, in patients, glass-ionomers interact chemically with hard tissues and this suggests that may indeed be considered bioactive.

## 1 Literature survey

The literature on materials widely regarded as bioactive, mainly glasses and ceramics, and glass-ionomer cements has been searched. The initial search was carried out using PubMed, and this was augmented by articles identified through Science Direct. Key words used were as follows: glass-ionomers; glass polyalkenoates; bioactivity; ion-release; interface; interaction zone; bioactive glass; mechanism of bioactivity; bioactive materials. In addition to the articles identified using these search tools, relevant papers already known to the authors have been included where they add insight and detail to the topic.

## 2 Introduction

So-called bioactive materials are becoming increasingly important for the repair of human tissues in a wide range of branches of medicine [1], including dentistry [2,3]. In general use, the term *bioactivity* has been applied to specific chemical substances, including vaccines, pharmaceuticals and food additives. When used for these substances, bioactivity is defined as “causing a reaction by, or triggering a response in, living tissue” [4,5].

Applying the term to materials used to repair parts of the body is less straightforward. However, there is growing effort to identify materials that can properly be described as bioactive, based on well-defined phenomena [3,6,7]. Such bioactivity generally implies that the interaction with natural tissue is beneficial and desired [2,3].

The original material described as “bioactive” was “Bioglass”, as developed by Professor Larry Hench and his group from 1969 [8]. Papers published as early as 1971 described this glass as *bioactive* [9], and the term was defined in this context as “the ability to form a mechanically strong bond between a host tissue and an implant” [8,9].

The first bioactive glass was based on four components, namely SiO_2_ (46.1 % by mass), CaO (26.9 %), Na_2_O (24.4 %) and P_2_O_5_ (2.6 %). It is known as Bioglass 45S5, and since it was first reported, it has found a number of uses in bone-contact applications [[10], [11], [12]]. It has the property of developing a hydroxyapatite carbonate layer, including partially crystalline hydroxyapatite, when soaked in simulated body fluid [13]. When used *in vivo*, this bioactive behaviour manifests itself in a strong interaction with bone cells, mainly osteoblasts, which are stimulated by the deposition of the initial calcium phosphate layer. This strong interaction results in a mechanically strong bond between the glass and the newly deposited bone [10,14].

Since the original bioactive glass composition was prepared, several other bioactive glasses have been fabricated and their biological action studied in detail. The majority have been based on the SiO_2_–CaO–Na_2_O–P_2_O_5_ system, but a number of other glass types, typically containing five or more components, have been considered [15]. Glasses have most often been fabricated by melting components, followed by quenching and milling to fine particle sizes. However, other fabrication methods, notably sol-gel synthesis, and quenching cooling to prepare porous glass particles, have also been used and studied in detail. In all cases, the glass in question is able to form a chemical bond with natural tissues, mainly bone, and this remains the criterion of bioactivity for these materials [16]. Studies have typically identified the compositional limits beyond which the glasses cease to show the property of bioactivity.

As well as bioactive glasses, a number of other materials are known to be bioactive in the same sense, i.e. because they promote bonding with natural tissues. These include the glass ceramic apatite-wollastonite (A/W glass ceramic), which will bond to bone, and has been commercialised for clinical use under the name of Cerabone® A-W [17]. Other bioactive materials include synthetic hydroxyapatite and β-tricalcium phosphate [18], both of which are capable of bonding to living bone in the same was as Bioglass [19]. These materials undergo similar surface reactions to bioactive glasses, *i.e.* release of calcium and phosphate ions into the surrounding body fluid, followed by precipitation of an amorphous calcium phosphate layer that can be colonised *in vivo* by osteoblast cells. This eventually leads to the development of fully functioning natural bone [20,21].

Calcium phosphate cements formed *in situ* are also considered bioactive [22]. These materials are prepared by mixing aqueous slurries of tetra-calcium phosphate and di-calcium phosphate, substances which react together to form hydroxyapatite [23]. The hydroxyapatite precipitates as a solid mass that includes all of the water in the original slurry [23]. The result is a weak but biologically active material that forms direct bonds with bone and can be used clinically to repair craniofacial defects and bone fractures [24,25].

Most bioactive materials are ceramics, but some metals and polymers are also bioactive. Neither type of material has been as extensively studied as ceramics, but both can interact positively with biological tissues if their composition and surface morphology are correct. As we have seen, the term “bioactive” was originally applied to speciality glasses, and their properties effectively defined the attributes necessary for a material to be considered bioactive. Given this, we will consider these properties in detail, and then examine the extent to which they are shown by glass-ionomer cements.

## 3 The concept of bioactivity

The term *bioactivity* was originally applied to materials such as speciality glasses to describe the positive response that occurs when the material is placed in the human body [1]. The most widespread current use of the term now typically includes:

- Release of biologically beneficial ions;
- Precipitation of a calcium phosphate layer;
- Stimulation of cell differentiation and proliferation.

The release of ions in itself is not enough for a material to be considered bioactive [2]. This is a necessary but not sufficient feature, and must be followed by the other two steps on this list. With certain materials, e.g. polymers, bioactivity may also involve the release of biological signalling molecules that promote restoration and repair of body tissues. However, this was not a feature of the original bioactive glasses, and not implied when the term “bioactive” was applied to these materials.

Developing a rigorous formal definition of *bioactivity* has received considerable attention over the years, mainly involving those materials which are used in contact with bone. Several similar definitions have been proposed, starting with an example agreed at a consensus conference held in Chester, UK, in 1987. The definition was:

*A material which has been designed to induce specific biological activity* [26].

Later Professor David Williams, a leading authority on biomaterials science then at the University of Liverpool, UK, suggested the definition:

*Phenomenon by which a material elicits or modulates biological activity* [27].

This is more extensive than the original definition, and no longer based on a single effect, namely the formation of a mechanically strong bond between the tissue and the implanted material. Rather, it can encompass a wide range of biological responses.

The term has also been defined with specific application to dentistry [26]. This occurred at the Northern Lights conference, held in Oslo in 2018 and the agreed definition states the following [28]:

*“Bioactivity” applied to a dental restorative material should describe an active beneficial biological process. It is suggested that dental restorative materials may be called “bioactive” if, in addition to their primary function of restoring or replacing missing tooth structure, they actively stimulate or direct specific cellular or tissue responses, or both, or they can control interactions with microbiological species*.

This is consistent with the use of the word *bioactivity* in other areas of biomaterials science, such as orthopaedics. However, there has since been a recent article which suggests that no dental restorative material fulfils this definition [29]. In fact, this assertion is not consistent with either the definition agreed in Oslo in 2018, or with other widely used definitions of the term. Similar recent articles [3,7] argue that the term as currently used is inappropriate. In the current paper we have striven to be careful about this and to use the term entirely in the way it was originally defined to describe the interaction of materials with biological systems. A criticism that the term “bioactive” is applied indiscriminately in the contemporary scientific literature [3] cannot reasonably be directed at the way we have used the term since we are using it to describe a specific type of interaction. Moreover, the term “bioactivity: has been applied to this type of interaction largely without challenge for over fifty years. This suggests, at the very least, that the designation is a useful one.

A notable feature of the current definitions and current usage is that the mechanism is not specified. The earliest materials described as *bioactive* functioned entirely due to their solution chemistry and had no active participation of cells until well into the process. Considering this behaviour in detail shows that some dental restoratives, in particular glass-ionomer cements, do show characteristics that may be considered to indicate bioactivity.

Last, there is a scientific journal called *Bioactive Materials* that has been published by KeAi Publishers in China since 2016. Its aims and scope, taken from the journal's website [30], includes the following definition of “bioactive materials”:

*Biomaterials that come into contact with cells, tissues and organs across all living species … designed to stimulate and/or direct appropriate cellular and tissue responses or control interactions with microbiological species* [30,31].

Here again there is no mention of the mechanism of bioactivity. Rather, the term “bioactive” used somewhat vaguely to cover a wide range of possibilities. Specifically, the word “bioactivity” does not imply any active involvement of cells, nor the release of biological signalling molecules. As a result, we must conclude that, from the usage throughout the scientific literature, the term *bioactivity* may involve a range of phenomena, and is not limited to any specific mechanism(s). However, it is still useful to consider the mechanisms displayed by bioactive glasses, and these are covered in the following section.

## 4 Mechanism of bioactivity

The reactions of bioactive glasses with body fluids has been studied extensively, and are able to explain to an extent why these substances are bioactive. The processes eventually involve bone bonding and formation of fully functioning bone and the later steps are complicated. However, the early stages are more straightforward and are reasonably well understood 108]. They involve a sequence that results in the formation of a layer of hydroxycarbonate apatite (HCA) the surface of the glass particles [32]. Once this layer is formed, the surface allows osteoblast cells to attach to it and to proliferate.

The early steps of this bioactivity process are [33]:

(i) Cation exchange in the presence of body fluids leading to the formation of silanol groups on the glass surface and the release of Na ^+^ ions:

-Si-O-Na^+^ + H^+^ → -Si-OH + Na^+^

(ii) Raising of the pH of the external solution as H^+^ ions are removed. This creates an imbalance in the amounts of H^+^ and of OH^−^ ions in which the latter predominate. This leads to an increase in pH.
(iii) Release of silica into the surrounding fluid, presumably as Si(OH)_4_. This re-polymerizes as -Si-O-Si- units at or near the glass surface.
(iv) The -Si-OH groups on the surface also react with Ca^2+^ ions, which in turn draw PO_4_^3−^ ions into the surface layer from the body fluid [34]. This causes a layer of amorphous calcium phosphate to form.
(v) Hydroxide and carbonate ions from the solution also become incorporated into the surface layer, and this eventually results in the formation of HCA [35,36].

Among the later more complicated steps leading to the formation of fully functioning bone are (a) adsorption of proteins onto the HCA surface, (b) attachment and differentiation of cells on this protein layer, and (c) production of bone matrix by the cells [2]. Together, these steps lead to the formation of complete bone which bonds strongly to the glass surface.

The earliest stages of the bioactivity process can be simplified to:

(i) Ion exchange between the glass surface and the surrounding body fluid;
(ii) Alteration of the local pH close to the glass surface, in the region called the “bioactivity zone” by Williams [37];
(iii) Precipitation of a new mineral phase (amorphous calcium phosphate) at the interface between the glass and body fluid;
(iv) Slow transformation of the mineral phase to HCA.

These steps are significant because similar processes occur when glass-ionomer cement is placed in contact with tooth tissues. These reactions, which have been described as “interfacial biomineralization” [6], involve no active biological processes and can take place *in vitro* in appropriate solutions with no cells present. Despite this, they are the steps that occur *in vivo*. In other words, this is a purely chemical process, but it is the means by which living bone and bioactive glass interact initially. It eventually goes on to generate the platform from which fully functioning bone, including bone cells, develops [38].

Other bioceramics are known to show similar behaviour. For example, apatite-wollastonite glass ceramic will develop a layer of calcium phosphate on exposure to simulated body fluid *in vitro* [39,40]. This behaviour mimics the observed *in vivo* action of this glass ceramic, where animal studies using rabbits have found that strong bonds form between the implant and living bone [41]. Also, hydroxyapatite itself shows similar behaviour [42,43]. Experimental studies have typically employed hydroxyapatite as a coating for metal implants, and reported that living bone grows right up against the HA coating, and forms a mechanically strong bond to it [43].

5. The interaction of glass-ionomers with biological tissues

This section is concerned with how glass-ionomer (glass polyalkenoate) dental cements behave under *in vivo* conditions, and in particular the similarity of this behaviour to that of bioactive glasses. Specific steps that occur on the interaction of glass-ionomers with natural tissues and fluids are considered, partly in the light of the recent helpful policy statement issued by the FDI (World Dental Federation) concerning the bioactivity of dental restorative materials [2]. Following this, some general conclusions are drawn.

Glass polyalkenoate dental cements are widely used clinically [44]. In their conventional form, they consist of a basic glass powder containing calcium (or strontium), sodium, silica, aluminium and phosphate ions, and a water soluble polymer such as poly(acrylic acid). The powder and liquid components are mixed before placement, and undergo an acid-base reaction to set within 2–3 min. The resulting rigid material has similar aesthetic properties to the natural tooth, and forms an adhesive bond to the tooth surface. These cements have been modified by the inclusion of a water-soluble monomer, typically 2-hydroxyethyl methacrylate. This monomer will undergo a polymerization reaction which augments the acid-base one, and provides a higher degree of control of the setting process for the clinician. The latter materials are known as resin-modified glass polyalkenoates (glass-ionomers). The following sections cover properties of both types of material, as both are capable of interacting with the natural tooth tissue.

(a) Release of ions

Both types of glass polyalkenoate cement have been shown to release a variety of ions, including sodium, aluminium, silicate, phosphate and fluoride in neutral conditions, plus either calcium or strontium in acidic conditions [6,[45], [46], [47]]. Most interest has been focused on fluoride because of its potential cariostatic action [48]. Release of fluoride is well established and has been known since the earliest days of these materials [49]. It can be sustained for considerable periods of time and occurs without any obvious damage to the remaining cement [50].

Release of fluoride is a two-step process. First comes the so-called early wash-out, a step which may actually take place for up to a month after the cement has been prepared [51]. Second, comes a long-term diffusion-based release [52] that may last for several years [50]. The nature of the second step depends on the pH of the storage solution; in neutral conditions it is a diffusion process, but in acidic conditions it is a slow dissolution/erosion process [51].

Not only does the mechanism of fluoride release change when the storage conditions change from neutral to acidic, so does the amount of fluoride released. In general, much more fluoride is released in acidic conditions than in neutral [46,48].

There are fewer published studies on the release of other ions from glass alkenoates [46,47], but results are quite clear. The ions Na^+^, Al^3+^, Si (as silicate) and P (as phosphate) are released under neutral conditions [46,47]. As with fluoride, greater amounts of these ions are released in acidic conditions than in neutral ones [47], and similar release behaviour is shown by resin-modified glass-ionomers [46]. There is also a change in the release profile, as under acidic conditions, either Ca^2+^ [46] or Sr^2+^ [53,54] is also released, depending on the formulation of the cement. Recent results have shown that sodium, aluminium, silicon and phosphorus have the same release kinetics as fluoride, i.e. two step release processes with early wash-out followed by either diffusion (in neutral conditions) or slow erosion (in acidic conditions) [55,56].

(b) Alteration of pH

Another feature of glass polyalkenoates, both conventional and resin-modified, which contributes to their bioactivity is that they alter the pH of the surrounding fluids [47,57,58]. When this effect was reported, it was termed *buffering* because it was assumed to arise from the presence within the cement of a partially neutralized weak acid (i.e. there were remaining acid functional groups) together with salts of that weak acid. These are the classic components of an acid buffer [59]. Recently, the buffer character of conventional glass polyalkenoates has been confirmed [60]. However, it is likely that any inherent buffer character is enhanced because considerable amounts of unreacted basic glass powder remain within the set cement, as this is capable of reacting with acidic external media to neutralize them.

Glass polyalkenoates retain the ability to alter the external pH for at least 6 months [61]. Any specific change in pH is rapid, and thin films of external lactic acid solution at pH 4.5 have been shown to be buffered to values around pH 5.5 within 30 s [62]. This has been considered clinically significant, since it corresponds to the change in pH on going from active to arrested caries [63].

These pH changes are associated with ion release [47]. However, the relationship is complicated, and successive identical changes in pH have been shown to be associated with wide variations in the relative amounts of ion released into the storage solutions. The importance of Na^+^ ions in this process has been demonstrated [47] and this is the same as for bioactive glass. This means that a similar mechanism underlies the pH change, *i.e.* H^+^ is exchanged for Na^+^, which causes the concentration of H^+^ to be reduced, thereby increasing pH.

As with bioactive glass, this ability to increase the external pH affects the solubility of a number of inorganic species. With bioactive glass, this causes amorphous calcium phosphate to precipitate, the first step in forming a surface for the deposition of fully functioning bone. Within the tooth, the increase in pH may also promote the deposition of calcium phosphate, and lead to the growth of the mineral phase known as the *interaction zone* [[64], [65], [66]].

Other species are released in acidic solutions, namely Ca^2+^ (or Sr^2+^), Al^3+^, phosphate and silicate [47]. The release of Al^3+^ has also been demonstrated in neutral conditions [67,68]. Release of Ca^2+^ and silicate mimics the behaviour of bioactive glasses [10,13,14] and, like those materials, may result in the formation of an active mineral layer, albeit one that does not have a composition identical to that of the natural mineralised tissue. Its occurrence, though, shows that the early steps in the interaction of glasses such as Bioglass 45S5 are strikingly similar to those of glass-ionomer cements with tooth tissues. They can therefore be considered as further evidence of the bioactivity of glass polyalkenoates.

(c) Mechanically strong bond formation to enamel and dentine

When ions are released from a glass polyalkenoate cement and exchanged with the surrounding tooth tissues, a distinct zone of interaction can be observed using SEM. Formation of this zone has been found both *in vivo* [[64], [65], [66]] and *in vitro* [69]. The *in vivo* results have been obtained with conventional glass polyalkenoate restorations and it has also been found that glass polyalkenoate fissure sealants cause an alteration of the tooth surface, which becomes “enamel-like” [70]. It probably arises from similar ion-exchange processes as the formation of the zone of interaction.

**Fig. 1:**
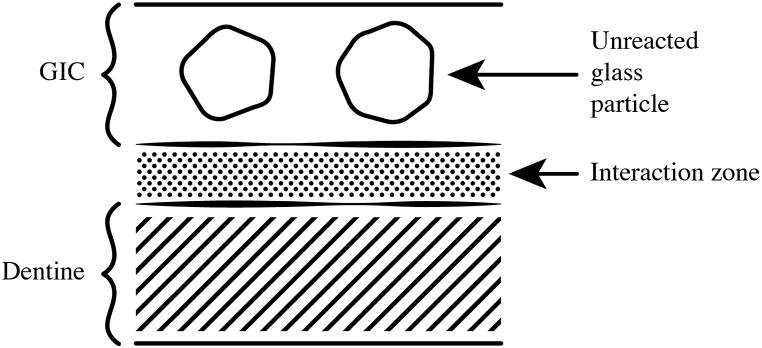
Diagram showing the zone of interaction formed between a tooth surface and a glass polyalkenoate restoration (based on observations by Ngo et al. [63]).

The initial study reporting the formation of this layer involved teeth that had been repaired with a cement which were removed after two years for orthodontic reasons. The zone of interaction between the tooth tissue and the glass polyalkenoate restoration was a clearly visible structure [65]. Later, a study was carried out using a low-temperature SEM technique to prevent the specimens drying out and cracking in the electron microscope. A diagram based on what was observed is shown as Fig. 1 [65]. This particular image is of a structure much younger than the ones originally observed, only 1–3 months old, but was still clearly visible. Analysis of the elemental composition showed it to be rich in ion-forming elements and to contain both strontium and calcium [65,71]. As the cement used in this study (Fuji IX GP) contains no calcium, but is strontium-based, the fact that the interaction zone contained both elements showed that it involved exchange of ions from both the cement and the tooth.

The formation of the ion-exchange layer is a purely chemical process and does not require vital teeth. This was demonstrated in a study with extracted teeth, where glass polyalkenoate restorations were placed then aged *in vitro* for up to a year [72]. Experiments showed that samples developed distinct zones of interaction that were the same as those in *in vivo* studies.

This has led some authors to propose the term “biointeractive” to describe these materials [2]. However, this term does not appear very help, especially as the layer formed was reported to be mechanically strong; however, no attempts were made to measure bond strength [65,66]. In fact, the measurement of bond strengths is complicated due to the weakness of glass polyalkenoate cements, which usually fail cohesively [72]. The absence of measurement of bond strength is similar to the situation with bioactive glass, where the phrase “mechanically strong” is used without quoting any numerical values. Whatever quantitative values might eventually be assigned, the fact that glass-ionomers form a bond with the tooth is completely analogous with bioactive glass and bone, and further evidence of glass polyalkenoates’ bioactivity.

As well as bonding to the tooth, conventional glass polyalkenoates have been shown to form direct bonds with bone. Initial studies were carried out some years ago at the time when there was interest in developing glass polyalkenoate bone cements based on conventional acid-base materials [73,74]. This led to a variety of *in vivo* experiments which showed that bone cells would migrate freely over the surface of freshly placed glass-ionomer cements, and eventually produce a strong cement-bone interface [[75], [76], [77]]. This implied that glass polyalkenoate bone cements were a possibility, and recently this interest has been revived [78]. A variety of bone-contact applications of conventional glass polyalkenoates have been proposed, not only for device fixation but also, more recently, for cranial fixation [79] and sternal fixation [80]. The latter was achieved using glass polyalkenoates of novel composition, i.e. based on tantalum-containing glasses [77].

These studies show that glass polyalkenoates have another feature shown by bioactive glasses, namely the ability to bond to bone without forming fibrous capsule. This is further evidence of their bioactivity. Recent studies using rabbit models have confirmed these earlier findings. Freshly placed cements showed a small early inflammatory response, after which they were well tolerated and became fully integrated into bone with a mechanically strong attachment [81].

(d) Demonstrating bioactivity *in vitro*

Within the field of bioactivity there is need to have an *in vitro* screening test to identify materials that are bioactive *in vivo*. The most widely used test is exposure of the material to the simulated body fluid (SBF) formulated by Kokubo [82]. Testing with this solution is now recommended in the appropriate ISO on the bioactivity of implants for surgery [83]. However, screening for bioactivity with this solution is far from straightforward and several papers have reported problems with it [[84], [85], [86]]. From the practical point of view the biggest drawback is that SBF can give both false positives and false negatives [85], though one report has suggested it gets it right in the majority of cases [86].

The original idea behind the development of simulated body fluid, SBF, seemed plausible. It was designed to have a composition resembling natural body fluids and is almost saturated in key ions, in particular Ca^2+^ and PO_4_^3−^. Release of appropriate ions from a material such as bioactive glass causes hydroxyapatite (or hydroxy-carbonate apatite) to precipitate from this solution. This substance can then be identified using FTIR, and its appearance is taken as evidence that the material is bioactive. In the case of many materials, including bioactive glasses and ceramics, this has been confirmed by experiments with cultured osteoblast cells and by *in vivo* experiments in animals [84]. However, for several materials, including glass-ionomers, the result from SBF is wrong. It fails to precipitate apatite onto the surface [87], despite the fact that, *in vitro*, the material will form a bond to hard tissues and show no indication of fibrous capsule.

The absence of a positive result from testing with SBF was first reported in 2001 by Kamitakahara et al. [87]. The result was attributed to the effect of poly(acrylic acid), which the authors suggested was released in small amounts from freshly mixed cements and then inhibits calcium phosphate precipitation. These authors noted that this was an anomaly, since even at that stage *in vivo* bone-contact experiments had shown that glass polyalkenoates were able to bond to living bone and hence were bioactive in the sense defined by Hench [9]. Evidence of this kind has been called for specifically by the FDI in order that a restorative material can be properly described as bioactive [2].

Since these initial studies, a number of papers have appeared reporting experiments where varying amounts of bioactive glass powders have been added to glass polyalkenoates to improve their bioactivity. These papers have tended to accept uncritically the results of testing with SBF and to report that, without bioactive glass, glass-ionomers are not bioactive. Studies have also shown that, when bioactive glass powder is added, amounts of calcium, silica and phosphate ions released increase [88], and to an extent that causes apatite to be deposited from SBF [89]. Without any added bioactive glass, neither conventional [90] nor resin-modified glass polyalkenoates [91] induce apatite precipitation in SBF. Despite this negative result in the screening test, glass polyalkenoates clearly cause a positive effect *in vivo* and are able to form mechanically strong bonds to the natural hard tissues dentine, enamel and bone. This is a result of their ion-release properties. In addition, using resin-modified glass-ionomers in cell cultures has shown that, even in the absence of bioactive glass powder, cells interact positively with the material. Cells attach readily to cement samples and proliferate on their surfaces [92].

## 5 Conclusions

Glass polyalkenoate cements, both conventional and resin-modified, may be counted as bioactive because they behave in a similar way to the speciality glasses and ceramics which are widely recognised as bioactive. The term *bioactive* has been applied to these latter materials for over 50 years, and is based on the following observations:

(i) They form a direct bond to living tissue (teeth and bones) without an intermediate layer of fibrous capsule;
(ii) They release specific ions that are able to promote the outcome in (i);
(iii) They alter the pH close to their surface, which promotes precipitation of calcium phosphate.

Glass polyalkenoates have been described as *bioactive* in the scientific literature for over twenty years but this bioactivity has not been widely recognised. One reason is that these materials do not promote the deposition of apatite from SBF. However, this finding has been explained in the literature, and is supported by *in vivo* results which show that there is distinct bioactivity in terms of positive interactions with natural tissues. These interactions are sufficient evidence of the bioactivity of glass polyalkenoates as demanded by the FDI's recent policy statement [2]. This bioactivity should be recognised by the dental profession and taken account of when selecting glass-ionomers for use clinically.

## Credit author statement

Beata Czarnecka: Writing – review & editing. Sharanbir Sidhu: Writing – review & editing. John Nicholson: Writing – review & editing, Writing – original draft, Conceptualization

## Data availability

All data are publicly available on the respective databases (PubMed and Science Direct).

## Declaration of competing interest

The authors declare the following financial interests/personal relationships which may be considered as potential competing interests:John Nicholson reports a relationship with GC Corp that includes: consulting or advisory. The other two authors declare that they have no known competing financial interests or personal relationships that could have appeared to influence the work reported in this paper.
